# Supplementary material for: Altered mental status is a predictor of poor outcomes in COVID-19 patients: A cohort study
Source: PLoS One. 2021 Oct 5;16(10):e0258095. doi: 10.1371/journal.pone.0258095 (PMC8491909; doi:10.1371/journal.pone.0258095)
Supplement: Attachment — Submitted filename: COVID-19-AMS.docx [file pone.0258095.s001.docx]

My comments are below:

Comment 1: In the line 81 and 82, 'Patients were divided into two groups: with and without altered mental status (AMS).' The definition of AMS was not clear. In order to get a more objective results, an operational definition for AMS is essential.

Comment 2:In the line 109 and 110, '...(BMI) was 33.32...with 57.2% being obese and 24.7 being overweight.' It's better to more clearly define 'obese' and 'overweight' . In addition, obesity is a critical issue for COVID-19 patients, it's also suitable to provide the BMI of normal population in New Orleans for comparison if possible.

Comment 3: The data of table 2 needs to be carefully corrected. For example, the meaning of the data in every parentheses made me confusing (e.g. in the first row of ED disposition : 134 (77) , 31 (72.1); however in the first row of Hospital admission: 310 (72.3), 41(57.7) ; the calculation method of each parentheses was inconsistent ! the inconsistency also noted in the sub-table of Procedures . Besides, the case numbers of hospital admission in Non-AMS group was 310+119=429 , but in the first row : Non-AMS (n=431), the authors should explain the discrepancy).

Comment 4:　In line 211-212 '... patients with AMS were less likely to be African American or have obesity...', but the authors didn't provide possible reasons for the differences. Besides, I wonder the body surface area might also differed between non-AMS and AMS groups.

Comment 5: According to fig 2, the AMS group was more likely to be intubated and needs mechanical ventilation, but in table 1, the respiratory symptoms of AMS group seemed to be less, especially ' Shortness of breath', this contradiction needs to be discussed.
