# Supplementary material for: Altered mental status is a predictor of poor outcomes in COVID-19 patients: A cohort study
Source: PLoS One. 2021 Oct 5;16(10):e0258095. doi: 10.1371/journal.pone.0258095 (PMC8491909; doi:10.1371/journal.pone.0258095)
Supplement: Attachment — Submitted filename: Response to reviewers.docx [file pone.0258095.s002.docx]

Response to reviewers' comments

**(PONE-D-21-02993)**

“**Altered mental status is a predictor of poor outcomes in COVID-19 patients: A cohort study.**”

Dear Prof. Emily Chenette,

Editor-in-Chief

PLOS ONE Journal

Thank you for giving me the opportunity to submit a revised draft of Our manuscript titled “**Altered mental status is a predictor of poor outcomes in COVID-19 patients: A cohort study**” to **PLOS ONE Journal**.

We appreciate the time and effort that you and the reviewers have dedicated to providing your valuable feedback on our manuscript. We are grateful to the reviewers for their insightful comments on our paper. We have been able to incorporate changes to reflect most of the suggestions provided by the reviewers. We have highlighted the changes within the manuscript.

Here is a point-by-point response to the reviewers’ comments and concerns.

| **Reviewer Comment** | |
| --- | --- |
| **Comment 1:** | In the line 81 and 82, 'Patients were divided into two groups: with and without altered mental status (AMS).' The definition of AMS was not clear. In order to get a more objective results, an operational definition for AMS is essential. |
| Author Response | Thank you for the comment. The Definition has been clarified in the manuscript and it is as following: altered mental status (AMS) which encompasses confusion, amnesia, loss of alertness, disorientation, defects in judgment or thought, unusual or strange behavior, poor regulation of emotions, and disruptions in perception, psychomotor skills, and behavior. Line (82-85) |
| **Comment 2:** | In the line 109 and 110, '...(BMI) was 33.32...with 57.2% being obese and 24.7 being overweight.' It's better to more clearly define 'obese' and 'overweight' . In addition, obesity is a critical issue for COVID-19 patients, it's also suitable to provide the BMI of normal population in New Orleans for comparison if possible. |
| Author Response | Thank you for pointing this out. We agree with this comment. Therefore, we have added the definition of the Obesity and overweight in the results. (line114,115)  The % of Obese population in Louisiana has been added to the discussion where our cohort had higher percentage of obese patient than the average of Louisiana population. Changes has been marked in the manuscript. (215-217) |
| **Comment 3(A):** | The data of table 2 needs to be carefully corrected. For example, the meaning of the data in every parentheses made me confusing (e.g. in the first row of ED disposition: 134 (77) , 31 (72.1); however in the first row of Hospital admission: 310 (72.3), 41(57.7) ; the calculation method of each parentheses was inconsistent ! the inconsistency also noted in the sub-table of Procedures . |
| Author Response | Thank you for pointing this out. The ED depositions data account for patients how came through the Emergency Room a total of 166 in non AMS and 43 for AMS and between the parentheses represent the (%) of the ER cases but these data don’t show any significance, so it was removed to avoid any confusion. (table 2) |
| **Comment 3(B)** | the calculation method of each parentheses was inconsistent! the inconsistency also noted in the sub-table of Procedures |
| **Author Response** | Thank you for pointing this out. The data has been clarified in the table caption. The data for the Extubation were calculated based on intubated patients and the no. of patients requiring reintubation were based on the no. of extubated patients. (Table 2) |
| **Comment 3(C)** | Besides, the case numbers of hospital admission in Non-AMS group was 310+119=429, but in the first row: Non-AMS (n=431), the authors should explain the discrepancy). |
| Author response: | Thank you for pointing this out. Revising our data showed it was a Clerical error. The correct no. is 431 in the Non-AMS group divided into Floor admission (312) and ICU (119) (table 2) |
| **Comment 4:** | In line 211-212 '... patients with AMS were less likely to be African American or have obesity...', but the authors didn't provide possible reasons for the differences.  B-Besides, I wonder the body surface area might also differed between non-AMS and AMS groups. |
| Author response: | A- Thank You for raising such an important point, however. Our Data could not explain why the AMS patients were less likely to be African or have obesity, but this strengthens the argument that AMS may be an independent risk factor for poor health outcomes in COVID-19 patients. This point was added as one of the limitations in the study to be addressed by future research. Line (235) B- We did not have access to the data for body surface area of the patients. So, we cannot address this point. |
| **Comment 5:** | According to fig 2, the AMS group was more likely to be intubated and needs mechanical ventilation, but in table 1, the respiratory symptoms of AMS group seemed to be less, especially ' Shortness of breath', this contradiction needs to be discussed. |
| **Author Response:** | We agree with this suggestion the contraindication could be explained due to the decreased reporting of presenting complain with AMS Patients according to  Han JH, Bryce SN, Ely EW, et al. The effect of cognitive impairment on the accuracy of the presenting complaint and discharge instruction comprehension in older emergency department patients. *Annals of emergency medicine* 2011;57(6):662-71.e2. doi: 10.1016/j.annemergmed.2010.12.002 [published Online First: 2011/01/29].  Amended in the discussion (199-201) |
