# Supplementary material for: Altered mental status is a predictor of poor outcomes in COVID-19 patients: A cohort study
Source: PLoS One. 2021 Oct 5;16(10):e0258095. doi: 10.1371/journal.pone.0258095 (PMC8491909; doi:10.1371/journal.pone.0258095)
Supplement: Attachment — Submitted filename: PLOS-COV-19-Revised.docx [file pone.0258095.s003.docx]

Comment 1:In the line 81 and 82, 'Patients were divided into two groups: with and

without altered mental status (AMS).' The definition of AMS was not clear. In order to

get a more objective results, an operational definition for AMS is essential.

Author Response Thank you for the comment. The Definition has been clarified in the

manuscript and it is as following: altered mental status (AMS) which encompasses

confusion, amnesia, loss of alertness, disorientation, defects in judgment or thought,

unusual or strange behavior, poor regulation of emotions, and disruptions in

perception, psychomotor skills, and behavior. Line (82-85)

**Responses 1: Although the authors described ‘altered mental status (AMS)’ in detail, it’s still difficult to practice in real world. The contents in Line (82-85) were only ‘descriptions’ of AMS, not ‘definitions’. In my opinion, it might be better to use the scores of Glasgow Coma Scale (GCS). In this way, we will be able to quantify AMS. It would also be easier for the audience to realize.**

Comment 2:In the line 109 and 110, '...(BMI) was 33.32...with 57.2% being obese and

24.7 being overweight.' It's better to more clearly define 'obese' and 'overweight' . In

addition, obesity is a critical issue for COVID-19 patients, it's also suitable to provide

the BMI of normal population in New Orleans for comparison if possible.

Author Response Thank you for pointing this out. We agree with this comment.

Therefore, we have added the definition of the Obesity and overweight in the results.

(line114,115)

The % of Obese population in Louisiana has been added to the discussion where our

cohort had higher percentage of obese patient than the average of Louisiana

population. Changes has been marked in the manuscript. (215-217)

**Responses 2: Agree.**

Comment 3(A):The dat of table 2 needs to be carefully corrected. For example, the

meaning of the data in every parentheses made me confusing (e.g. in the first row of

ED disposition: 134 (77) , 31 (72.1); however in the first row of Hospital admission:

310 (72.3), 41(57.7) ; the calculation method of each parentheses was inconsistent !

the inconsistency also noted in the sub-table of Procedures .

Author Response Thank you for pointing this out. The ED depositions data account for patients how came through the Emergency Room a total of 166 in non AMS and 43 for AMS and between the parentheses represent the (%) of the ER cases but these datadon’t show any significance, so it was removed to avoid any confusion. (table 2)

**Response 3(A): Agree.**

Comment 3(B)the calculation method of each parentheses was inconsistent! the

inconsistency also noted in the sub-table of Procedures

Author Response Thank you for pointing this out. The data has been clarified in the

table caption. The data for the Extubation were calculated based on intubated patients and the no. of patients requiring reintubation were based on the no. of extubated patients. (Table 2)

**Response 3(B): Agree.**

Comment 3(C)Besides, the case numbers of hospital admission in Non-AMS group

was 310+119=429, but in the first row: Non-AMS (n=431), the authors should explain

the discrepancy).

Author response:Thank you for pointing this out. Revising our data showed it was a

Clerical error. The correct no. is 431 in the Non-AMS group divided into Floor admission (312) and ICU (119) (table 2)

**Response 3(C): Floor admission was still 310 in table 2. This should be corrected.**

Comment 4: In line 211-212 '... patients with AMS were less likely to be African

American or have obesity...', but the authors didn't provide possible reasons for the

differences.

B-Besides, I wonder the body surface area might also differed between non-AMS and

AMS groups.

Author response:

A-Thank You for raising such an important point, however. Our Data could not explain

why the AMS patients were less likely to be African or have obesity, but this

strengthens the argument that AMS may be an independent risk factor for poor health outcomes in COVID-19 patients. This point was added as one of the limitations in the study to be addressed by future research. Line (235)

**Response 4(A): Agree.**

B-We did not have access to the data for body surface area of the patients. So, we

cannot address this point.

**Responses 4(B): Agree.**

Comment 5:According to fig 2, the AMS group was more likely to be intubated and

needs mechanical ventilation, but in table 1, the respiratory symptoms of AMS group

seemed to be less, especially ' Shortness of breath', this contradiction needs to be

discussed.

Author Response:

We agree with this suggestion the contraindication could be explained due to the

decreased reporting of presenting complain with AMS Patients according to

Han JH, Bryce SN, Ely EW, et al. The effect of cognitive impairment on the accuracy of

the presenting complaint and discharge instruction comprehension in older emergency department patients. Annals of emergency medicine 2011;57(6):662-71.e2. doi:10.1016/j.annemergmed.2010.12.002 [published Online First: 2011/01/29].Amended in the discussion (199-201)

**Responses 5: Agree**.
