# Supplementary material for: Altered mental status is a predictor of poor outcomes in COVID-19 patients: A cohort study
Source: PLoS One. 2021 Oct 5;16(10):e0258095. doi: 10.1371/journal.pone.0258095 (PMC8491909; doi:10.1371/journal.pone.0258095)
Supplement: Attachment — Submitted filename: Response to reviewers.docx [file pone.0258095.s005.docx]

Response to reviewers' comments

**(PONE-D-21-02993)**

“**Altered mental status is a predictor of poor outcomes in COVID-19 patients: A cohort study.**”

Dear Prof. Emily Chenette,

Editor-in-Chief

PLOS ONE Journal

First, we apologize for delayed response because of hurricane Ida and the power loss afterward. we are extremely grateful for both reviewers’ comments and feedback. We would like to thank the editors and reviewer for giving us the opportunity to submit a revised draft of Our manuscript titled “**Altered mental status is a predictor of poor outcomes in COVID-19 patients: A cohort study**” to **PLOS ONE Journal**.

We appreciate the time and effort that you and the reviewers have dedicated to providing your valuable feedback on our manuscript. We are grateful to the reviewers for their insightful comments on our paper. We have been able to incorporate changes to reflect most of the suggestions provided by the reviewers. We have highlighted the changes within the manuscript. We hope that our responses address the reviewers concerns and wish it meets PLOS journal standards.

Here is a point-by-point response to the reviewers’ comments and concerns.

| **Reviewer 2 Comments** | |
| --- | --- |
| **Comment** | I suggested the authors to provide the Glasgow Coma Scale (GCS) score of the AMS patients previously. However, I still cannot get any GCS data by this revised article. I think this point needs to be well explained. |
| **Answer** | Thank you for your comments and reviews, we have edited the methodology section to show how used GCS and edited the results to highlight this finding  Line 91-92  Line 129-130 |
